# Effect of Flow State of Pure Aluminum and A380 Alloy on Porosity of High Pressure Die Castings

**DOI:** 10.3390/ma12244219

**Published:** 2019-12-16

**Authors:** Hanxue Cao, Chengcheng Wang, Junqi Che, Ziwei Luo, Luhan Wang, Lang Xiao, Jing Wang, Tao Hu

**Affiliations:** 1 College of Materials Science and Engineering, Chongqing University, Chongqing 400044, China; cjq342561227@163.com (J.C.); luo51520339@163.com (Z.L.); 201709131164@cqu.edu.cn (L.W.); 17623531908@163.com (L.X.); wangjing30353834@163.com (J.W.); 20173003@cqu.edu.cn (T.H.); 2 National Engineering Research Center for Magnesium Alloys, Chongqing University, Chongqing 400044, China; 3 Chongqing Automotive Collaborative Innovation Center, Chongqing University, Chongqing 400044, China; 13883258439@163.com

**Keywords:** die-casting filling, real-time observation, air entrapment, porosity

## Abstract

Air entrapment defects prevent the heat treatment from improving the mechanical properties of die castings, which limits the die casting of high-performance components. The flow pattern of the filling process is complicated and experimental analysis is difficult in thin-walled complex die castings. In this study, we constructed a shock absorption tower to observe in real-time the filling process of pure aluminum and A380 aluminum alloy at different fast injection speeds. The degree of breakup of pure aluminum was larger than that of A380 during the filling process, which caused the porosity of pure aluminum to be greater than that of the A380 at each observation position. Re-Oh diagrams explained the difference in porosity between the two metals. The porosity in different regions was closely related to the flow state of aluminum liquid. In addition to porosity measurements, we specifically analyzed the relationship between the porosity of the flowback zone, the final filling zone, and the near-tail zone of cylinder. At the same injection velocity, the porosity at flowback zone was greater than that at the final filling position, the porosity at final filling position was larger than that at the near-tail zone of cylinder, and the final filling position changed as the injection velocity changed.

## 1. Introduction

High pressure die casting (HPDC), which is a special type of casting method, has been widely used in automotive fields due to its high productivity and its excellent dimensional and shape accuracy [1]. Gas-induced porosity is the main limitation and important problem in HPDC [2]. The filling time is extremely short, gas trapped during the high-speed injection causes formation of pores, because of the characteristics of “high-speed filling” of die casting [3,4]. Porosity affects the conventional heat treatment properties of castings, thus degrading the quality of castings [5]. The production of die casting heavily relies on experience. The theory and technology of die casting are still immature, especially the actual filling process and gas porosity distribution of die casting. Thus, it is of great engineering and theoretical value to study the die casting filling process and to predict the distribution of gas entrapment to improve the performance of die castings. This research will also significantly promote the development of lightweight automotive technology.

Currently, the popular ways of studying the die casting filling process and predicting gas entrapment are computational fluid mechanics and experimental fluid mechanics [6].

The filling process is a typical two-phase flow (gas-liquid) [7,8]. Niu et al. investigated a new simulation program for gas-liquid two-phase mold filling [7], while using the Level set method to track the gas-liquid interface boundary, and simulated a benchmark filling experiment. The investigators showed that the program properly predicted the gas-liquid two-phase mold filling process in casting.

Researchers often choose a water analogue experiment to study the die casting filling process and verify numerical predictions [9,10,11,12,13,14]. Cleary et al. tested the prediction of a smoothed particle hydrodynamic (SPH) model in characterizing the filling process [9]. They used water simulation experiments to verify the SPH model predictions of thin-walled casting. Yuan et al. studied the flow patterns of liquid metal in the injection cavity by establishing a three-dimensional (3D) computational fluid dynamics model [10]. They designed a water simulation system for investigating the slow injection process of die casting. Yuan et al. verified the numerical model of injection processing in the cold chamber by comparing numerical simulation results with water simulation experimental results. According to Chimani et al., the global spreading of the free jet in the casting mold was well envisioned by this first numerical simulation that used water modeling to validate the numerical results [11]. The flow characteristics were compared with product quality results in Al pressure die casting parts of similar design.

Shahane et al. studied a new computational framework for simulating the heat transfer, solidification, and fluid flow in casting processes [15]. Fu, et al. studied the the effects of input uncertainty on the outputs in HPDC simulations, and carried out three uncertainty propagation experiments to research the impact of uncertainty in metal material properties and the thermal boundary conditions [16]. Han et al. studied a variable Spacing Even Mesh (VSEM) method, which was proposed to integrate with a computational fluid dynamics technique, SOLAMAC, to simulate the flow pattern in the shot sleeve [17]. They tested the model on the shot sleeve of a cold chamber die casting machine to demonstrate the effects on the flow pattern of molten metal in the shot sleeve. Cao et al. used the gas-liquid multiphase flow model to research the prediction of gas entrapment defects in Zinc Alloy HPDC [3]. They used the continuum surface force (CSF) model to treat the surface tension of gas-liquid multiphase. Cao et al. used a water-filling experiment that was simulated in an S-shaped channel, and the simulation results were closely consistent with the experimental results, which indicated the accuracy of the model. However, from the principle of a water simulation experiment, similar strictly dynamic conditions are difficult to achieve in the actual situation, that is, it is difficult to fully realize the equality of dimensionless criteria. In addition, liquid metal will undergo heat transfer and violent collision with the mold in the actual die casting filling process. Especially during the filling of complex thin-walled die-casting parts, the viscosity and other characteristics will change as temperature decreases. Additionally, the physicochemical properties of water and liquid metal are very different, so the application of a water simulation experiment has certain limitations, and it is more suitable for the filling of a die casting with a simple structure.

Real-time X-ray radiography is another popular way of observing the filling process in casting. Griffiths and Ainsworth used real-time X-ray radiography to investigate the nature of the liquid metal-pattern interface during mold filling in Lost Foam casting of aluminum alloys [18]. They found that the advancing liquid metal front became unstable above a certain critical velocity, which leads to an entrainment of the degrading pattern material and associated defects. Ohnaka et al. used X-ray imaging to observe the actual melt die casting process [19]. By comparison with the numerical simulation results, they found that the surface tension of the molten metal was critical in the accuracy of the numerical simulation results. In addition, they investigated a way of eliminating air entrapment. X-rays have weak penetration for steel molds, although X-ray technology can realize real-time observation of the die casting filling process, thus making imaging difficult. It is necessary to use special die casting molds, such as graphite molds [19], and, even then, the image is blurred. In addition, real-time X-ray imaging entails harsh operating conditions and it requires expensive equipment, which hinders wide applicability.

In this paper, we introduce a new method to observe directly the actual filling process in HPDC. We performed six flow visualization experiments. We used a shock absorption tower to observe the pure aluminum and A380 aluminum alloy during the filling process at different fast injection speeds. Being combined with porosity measurements, we analyzed and predicted the locations and size of air entrapment defects. We specifically analyzed the relationship between the porosity of the flowback zone, the final filling zone, and the near-tail zone of cylinder.

## 2. Experiments

An experimental study was performed while using a horizontal cold chamber die casting machine (Buhler Evolution machine built by L.K. Technology Holdings Limited, Hong Kong, China). The casting materials were 99.7% pure aluminum and A380 aluminum alloy (Table 1). X-ray fluorescence spectrometer (XRF-1800 built by Shimazu Enterprise Management Limited, Beijing, China) determined the compositions of the alloy in Chongqing University. Six flow visualization experiments were conducted, and Table 2 shows their specific parameters. The first fast shot point was 150 mm, 270 mm for the second fast shot point, and 357 mm for the maximum shot point. The mold preheating temperature was 200 °C. The nominal pressure of the Buhler Evolution machine was 13.5 MPa. There was no pressure intensification stage in the experiments. The inner diameter of the elastic sleeve was 70 mm and the length was 380 mm.

**Figure 1:**
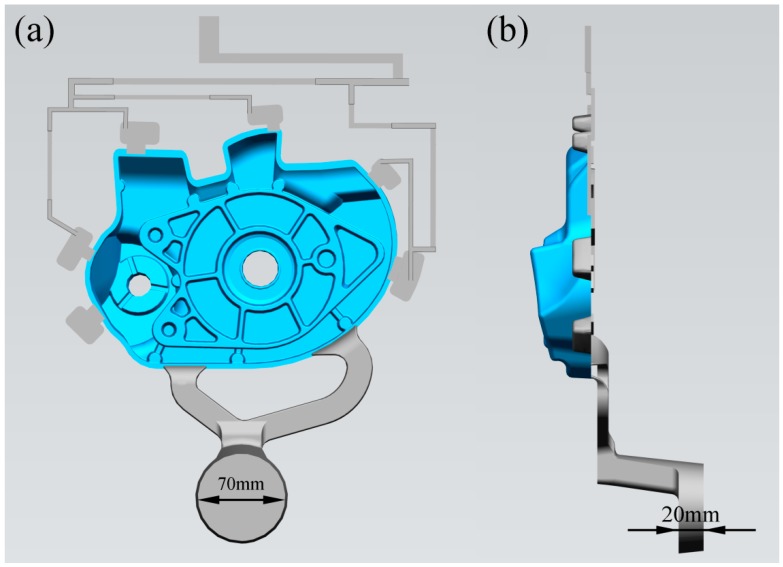
Schematic of die casting geometry for the flow visualization experiment [20]. (**a**) vertical view; (**b**) side view.

**Figure 2:**
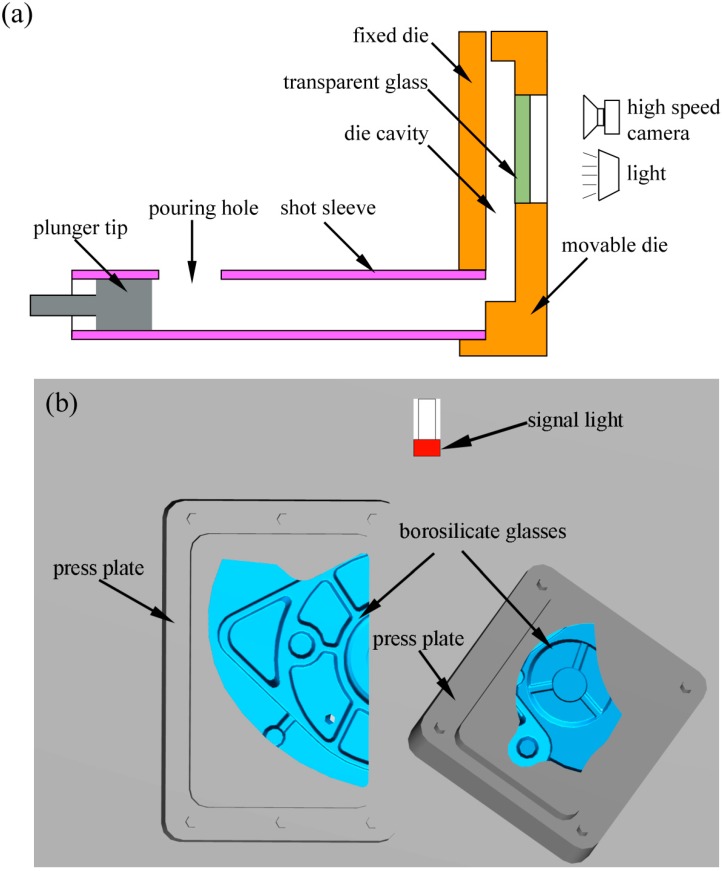
Schematic of the flow visualization setup. (**a**) The experimental setup; and, (**b**) Diagram of two shooting windows with a high speed camera (large size 150 mm × 101 mm, small size 104 mm × 79 mm) on a movable mold [20].

Figure 1 shows a schematic of the die casting geometry for the flow visualization experiment. The size of the shock absorber is 250 × 194 × 49 mm, the average wall thickness is 4 mm, the volume is 303,030 mm^3^, and the projected area is 127,723 mm^2^. The gating system uses double ingates, the total area of the ingate is 192 mm^2^, and the diameter of the sprue is 70 mm. Figure 2 shows the schematic of the flow visualization setup. The transparent borosilicate glass (Table 3) was placed on a movable mold to observe the flow law in real time during die casting. Figure 2b shows the position and size of two transparent windows (large size 150 mm × 101 mm, small size 104 mm × 79 mm), which were parallel to the casting. The left window was a vertical surface and the bottom was located at the inner gate, which was convenient for observing the flow of aluminum liquid into the cavity. In the right window, there were two staggered cylinders, and it was essential for studying the flow of liquid metal around the cylinders in HPDC. We captured the flow pattern of the aluminum melt with a high-speed camera at 1000 frames per second sampling rate and 1/1000 s shutter speed. Figure 2b shows a signal light that was placed in the shooting area to confirm the start time of plunger tip movement. The signal light became illuminated as soon as the plunger head began to move.

**Figure 3:**
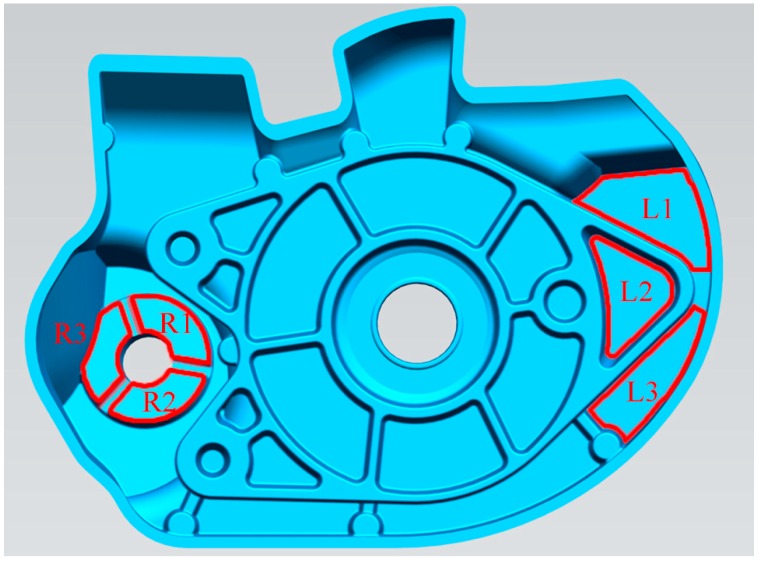
Schematic of sample location (L1, L2, and L3 in the left window, and R1, R2, and R3 in the right window).

Figure 3 shows the sampling locations of the two kinds of liquid. We calculated the porosity of the casting was evaluated, according to the standard BN75/4051-10 [21]. The density was measured while using the Archimedes method, and the alloy density was calculated by Equation (1).

(1) $$\rho_{p}=\frac{m_{1}}{m_{1}-m_{2}}\cdot \rho_{w}$$

where $m_{1}$ is the mass of specimen in air, $m_{2}$ is the mass of specimen in water, $\rho_{p}$ is the density of specimen, and $\rho_{w}$ is the density of water.

Next, Equation (2) calculated the specimen porosity.

(2) $$P=\left(1-\frac{\rho_{p}}{\rho_{wz}}\right)\times 100\%$$

where $\rho_{wz}$ is the true density, which is 2705 kg/m^3^ for pure aluminum and 2740 kg/m^3^ for the A380 aluminum alloy.

The samples were analyzed for microstructure. X-ray detection equipment UNC130 (Shenzhen Unicomp Technology Co., Ltd, Shenzhen, China) was used to locate the pore defects. The optical microscopy of the microstructure and pore defects were performed in the Central Laboratory.

**Figure 4:**
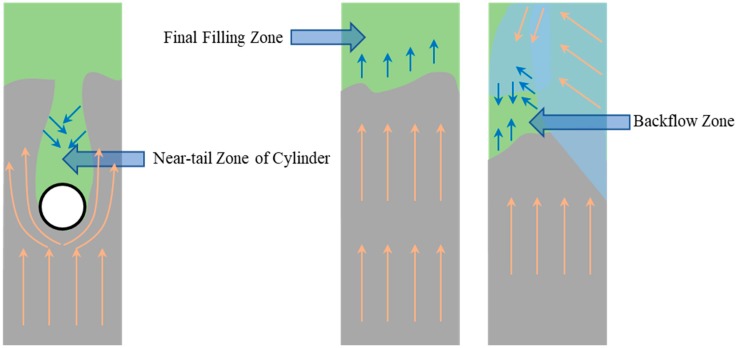
Schematic of three typical alloy filling flow patterns.

We observed three typical alloy filling flow patterns, as shown in Figure 4, backflow zone, near-tail zone of cylinder, and final fillinf zone, respectively.

## 3. Results and Disscussion

### 3.1. Comparison of Pure Aluminum and A380 Aluminum Alloy Filling Process

**Figure 5:**
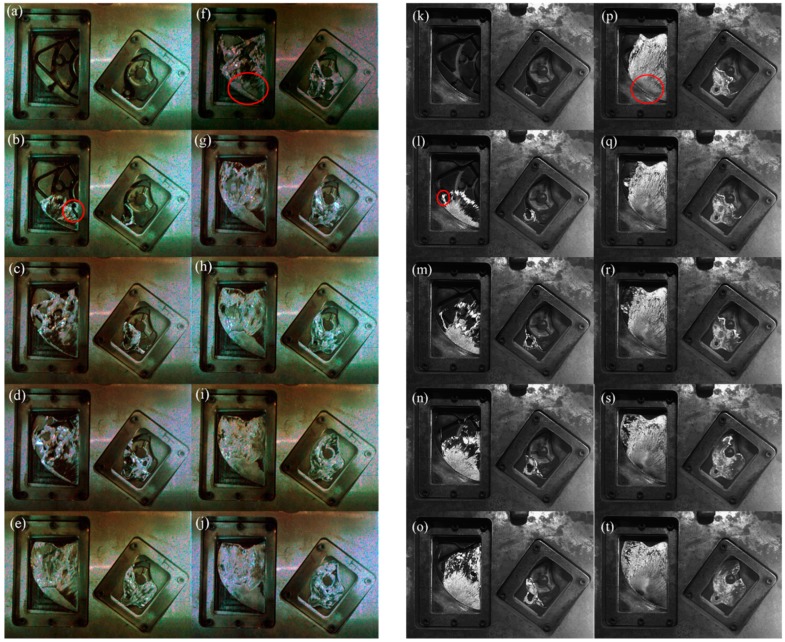
Observations of filling process for pure aluminum (**a**–**j**) and A380 aluminum (**k**–**t**).

We compared the A1 experiment (Table 2) at 0.88 m/s to the B2 experiment (Table 2) with a similar injection velocity of 0.80 m/s to compare the actual filling process of pure aluminum and A380 aluminum alloy. Figure 5 shows the comparison of observations for pure aluminum (left) and A380 aluminum (right) within the transparent windows.

Figure 5a,k show the fluid entering from the right window. In the right windows of Figure 5b,l, the liquid aluminum met the raised cylinder and began to flow around it, but the shear layer that was separated by pure aluminum from the cylinder was longer. In Figure 5b, a stream of pure aluminum melt from the ingate on the right entered the left window (circled in red in the Figure).

As shown in Figure 5c,m, the flow states of pure aluminum and A380 were significantly different. We observed cracks in both the left and right windows in the pure aluminum liquid. The A380 melt also cracked, but it quickly returned to a continuous state and only broke in the left window.

Figure 5f,p show the two-fast shot phase and a high-speed jet (shown in a red circle) that appeared in the left window. The pure aluminum was still in a ruptured state, whereas the A380 aluminum alloy appeared to be continuous.

Overall, from the entire filling process, the differences between pure aluminum and A380 aluminum alloy were mainly caused by the degree of cracking of continuous aluminum liquid. Pure aluminum had more rupture during the filling process, whereas the A380 aluminum alloy had a small degree of rupture and it remained substantially continuous.

**Figure 6:**
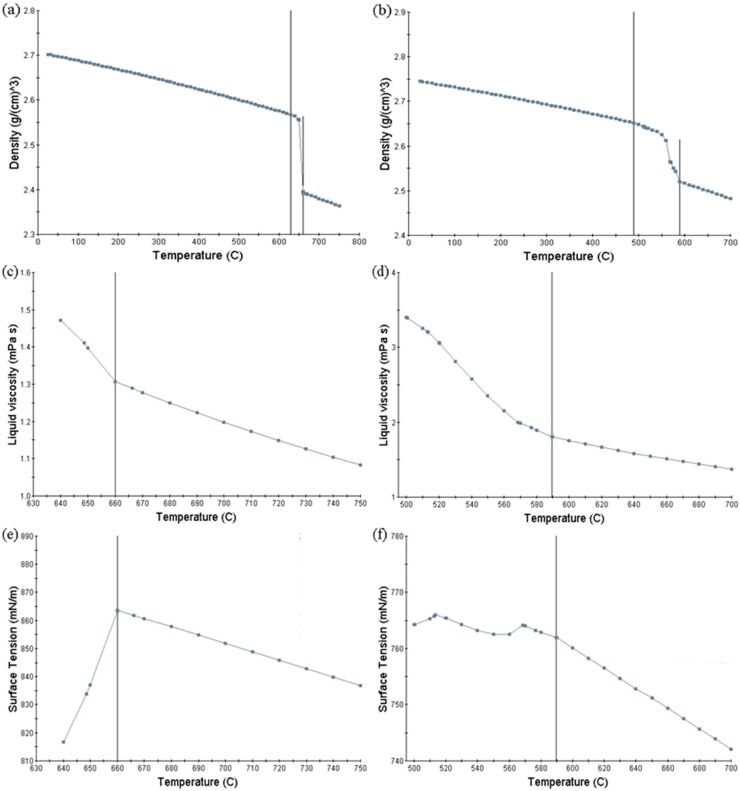
JMatPro calculations. (**a**) the density of pure aluminum; (**b**) the density of A380 aluminum alloy; (**c**) the viscosity of pure aluminum; (**d**) the viscosity of aluminum alloy A380; (**e**) the surface tension of pure aluminum; and, (**f**) the surface tension of A380 aluminum alloy.

The Ohnesorge number is a dimensionless number that measures the relationship between viscous forces and inertial forces and surface tension; it is an important parameter in characterizing fluid fracture decomposition [22].

(3) $$Oh=\frac{\sqrt{We}}{Re}$$

(4) $$We=\frac{\rho u^{2}d}{\sigma }$$

where *We* is the Weber number, which is used to describe the importance of the fluid inertial force relative to the surface tension; *Re* is the Reynolds number, which can characterize the energy provided at the ingate; ρ is the fluid density (kg/m^3^); *d* is the thickness of the gate, *d* = 2.5 × 10^−3^ m; u is the velocity of the aluminum liquid at the ingate (m/s); *d* is the thickness of the gate, *d* = 2.5 × 10^−3^ m; and, *σ* is the fluid surface tension (N/m). JMatPro (10.0, Sente Software Ltd., Guildford, UK) calculated the density, viscosity, and surface tension of pure aluminum and A380 and Figure 6 shows the calculation results.

**Figure 7:**
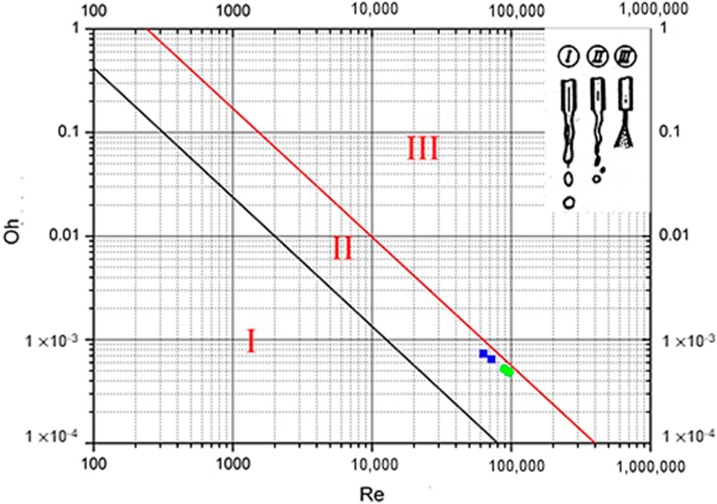
Three regimes of droplet formation from the jet (I: splash, II: wavy disintegration, III: atomization) [23]. The green circle corresponds to pure aluminum at 750 °C and 710 °C. The blue square corresponds to A380 aluminum alloy at 700 °C and 660 °C.

Table 4 shows the *Oh*, *We*, and *Re* numbers at the gates of pure aluminum at 750 °C and 710 °C, and the A380 aluminum alloy at 700 °C and 660 °C. Energy is required to turn the jet bursts into droplets. The energy that is required is proportional to the surface tension, and the *Oh* number can characterize the relative magnitude of the surface tension because the surface area increases during droplet formation. In addition, the necessary energy can be provided by the ingate or the shear forces acting on the jet, and the energy that is provided at the ingate can be characterized by Re. Therefore, the size and formation of these droplets can be characterized by Re and the *Oh*, and the formation can be divided into three main states: (I) splattering; (II) wavy disintegration; and, (III) atomization [10], as shown in Figure 7.

Figure 7 shows the state of pure aluminum under the A1 experimental conditions and the A380 aluminum alloy under the B2 experimental conditions. Pure aluminum and A380 aluminum alloy were both in the II state: wavy disintegration. Therefore, pure aluminum under A1 conditions and the aluminum alloy under B2 conditions each had an aluminum liquid rupture phenomenon and no atomization phenomenon. The pure aluminum liquid under A1 was closer to the boundary line of the II and III regions than was the A380 aluminum alloy under B2, so the pure aluminum liquid was more likely to be broken.

**Figure 8:**
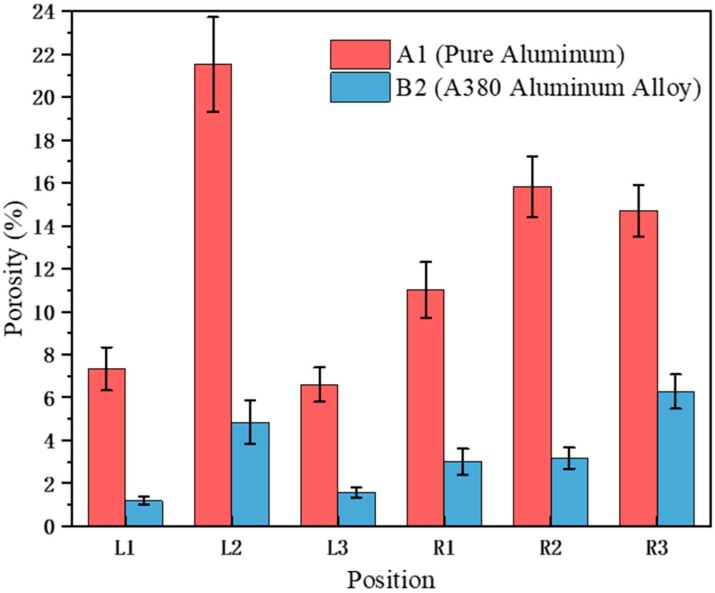
Porosity of different positions of pure aluminum (A1) and A380 aluminum alloy (B2).

Figure 8 shows the porosity of pure aluminum under the A1 conditions and the A380 aluminum alloy under the B2 conditions. The porosity of pure aluminum was significantly greater than the porosity of the aluminum alloy at each position. The greater porosity of pure aluminum was mainly due to cracking during the filling process, which presented significant turbulence and greatly increased the possibility of gas entrapment. In addition, the rupture of the continuous aluminum liquid will increase the surface area, thereby increasing the oxide film area. These oxide films can become heterogeneous nucleation sites for bubbles, which will greatly promote the formation of pores [24]. The density variation between solid and liquid for pure Al was higher than A380, and shrinkage porosity could be reasons for higher porosity for pure Al, especially in backflow regions, according to JMatPro data. On the one hand, the A380 aluminum alloy contains a certain amount of Si, which reduces its pore shrinkage porosity, on the other hand, there is not enough liquid metal that is available to compensate for the volume contraction of the solidifying region in backflow region, and the shrinkage porosity defects will form.

### 3.2. Effect of Injection Velocity on Melt Flow Pattern and Gas Defects of A380

#### 3.2.1. Porosity Prediction in the Left Window

**Figure 9:**
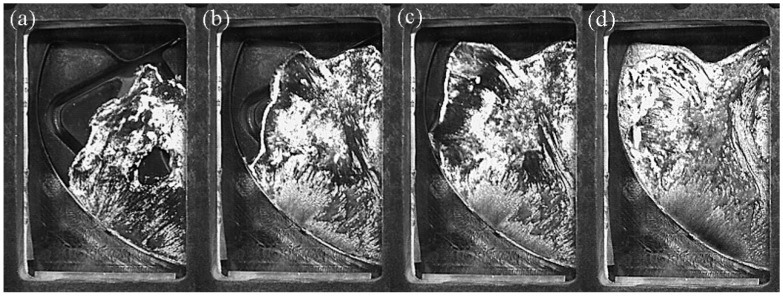
Flow pattern of A380 aluminum alloy melt at a fast shooting velocity of 0.66 m/s in the left window. (**a**–**d**): Filling status at different filling times.

Figure 9 shows the flow pattern of A380 aluminum alloy melt at a fast injection velocity of 0.66 m/s in the left window. L1 is the final filling zone. Figure 9c shows that the liquid metal smoothly advanced from the ingate to the left side of the window, first filling the L2 and L3 regions, and finally reaching the L1 region.

L2 is the flowback zone. In Figure 9d, the aluminum liquid was affected by the top of the mold cavity and the cylindrical boss on the right in the left window. A counterclockwise backflow zone was formed on the left side of the window, and its center was located in the L2 region. L3 represents the other zone.

**Figure 10:**
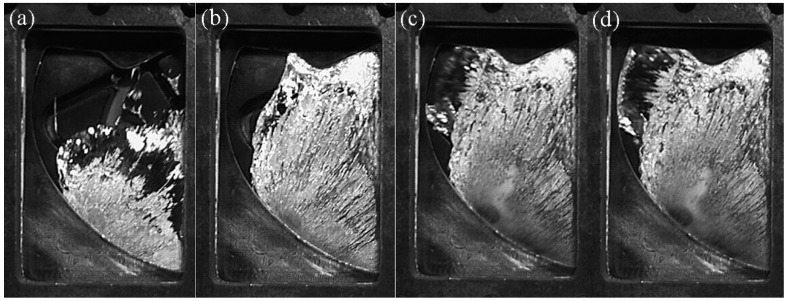
Flow pattern of A380 aluminum alloy melt at the fast shooting velocity of 0.80 m/s in the left window. (**a**–**d**): Filling status at different filling times.

Figure 10 shows the flow pattern of the A380 aluminum alloy melt at the fast injection velocity of 0.80 m/s in the left window. The flow state was similar to the flow state at a fast injection velocity of 0.66 m/s. The change of fast injection velocity caused a change in the final filling zone. In Figure 10d, the L1 and L2 positions were first filled, and the L3 region was the final filling position.

**Figure 11:**
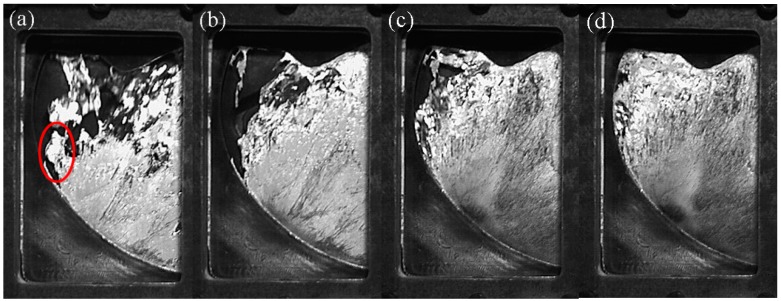
Flow pattern of A380 aluminum alloy melt at the fast shooting velocity of 1.06 m/s in the left window. (**a**–**d**): Filling status at different filling times.

Figure 11 shows the flow pattern of A380 aluminum alloy melt at the fast injection velocity of 1.06 m/s in the left window. L1 was the final filling zone, L2 was the flowback zone, and L3 was the other zone in this filling process.

**Figure 12:**
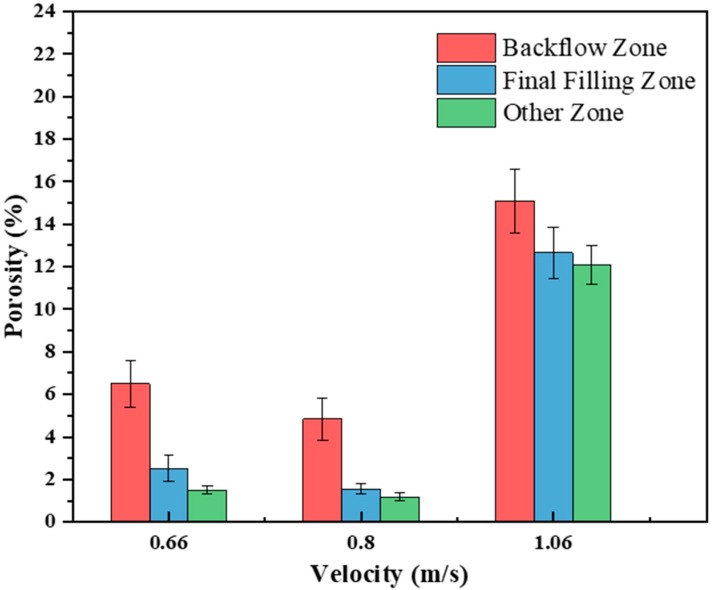
The porosity at different locations in left window with different fast shooting velocities (0.66 m/s, 0.8 m/s, and 1.06 m/s).

Figure 12 shows the porosity at different positions of the A380 aluminum alloy die casting with different fast injection speeds. The flowback zone had the highest porosity, followed by the final filling zone. The vortex formed in the backflow zone, the center of which was the low-pressure zone. Low-density dissolved or free gases were absorbed into the backflow zone, thereby increasing the porosity. In addition, the backflow zone was far away from the wall surface and the ingate, and the heat transfer was slow, thus the backflow zone became the final solidification area where it was easy to generate more shrinkage and shrinkage holes.

The aluminum liquid was obviously broken in Figure 11a (red circle). The breaking of the liquid occurred mainly, because, as the injection velocity increases, the *We* number increases, and the surface tension is relatively reduced, so that the possibility of liquid aluminum cracking is increased. The aluminum liquid began to flow toward the 11 o’clock direction after colliding with the top of the mold cavity and the cylindrical boss on the right side of the left window. The interface length of the flow front was long, but the flow front was uneven and rough (Figure 11c). The rough flow front will substantially increase the amount of entrapped gas, so that the porosity increased overall at a fast injection speed of 1.06 m/s.

**Figure 13:**
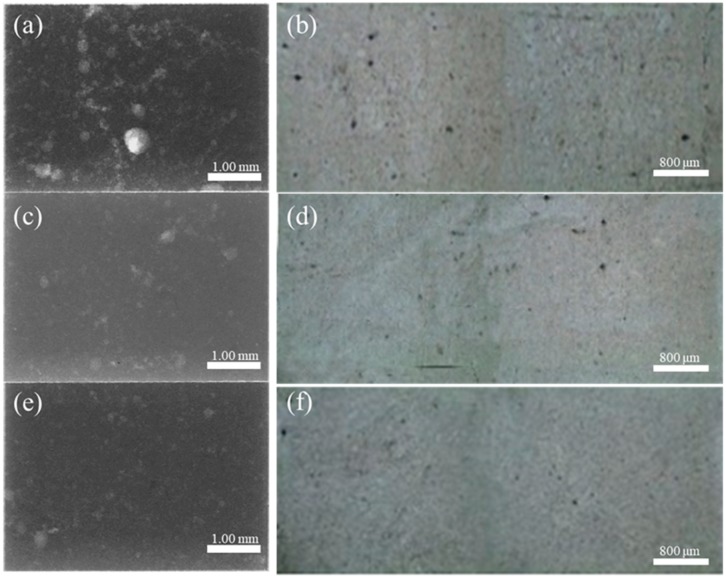
Comparison of porosity microstructure (**a**) X-ray image of backflow zone; (**b**) OM image of backflow zone; (**c**) X-ray image of final filling zone;(**d**) OM image of final filling zone; (**e**) X-ray image of other zone; (**f**) OM image of other zone.

Figure 13 shows the microstructures, and defects (porosity) of specimens from backflow zone, final filling zone, and other zone under the velocity of 0.8 m/s, the white highlights in X-ray images are pore defects. We combined two 50× microscope images to compare the porosity defects of three specimens. The pores in the flowback zone are larger and more numerous, which has the same conclusion as the calculation.

#### 3.2.2. Porosity Prediction in the Right Window

**Figure 14:**
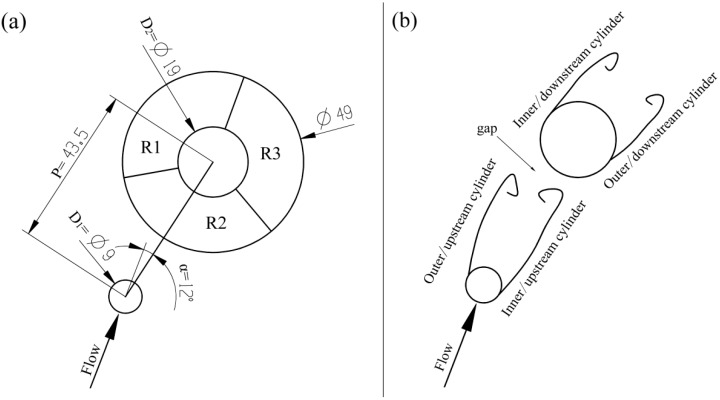
(**a**) Schematic of the arrangement of the two cylinders in the right window; and, (**b**) Schematic of shear layer designations [20].

The flow in the right window can be simplified as a plane flow around the two misaligned cylinders, according to the flow state of the aluminum liquid in the video obtained by direct observation. Figure 14 shows the configuration of the two cylinders in the right window.

**Figure 15:**
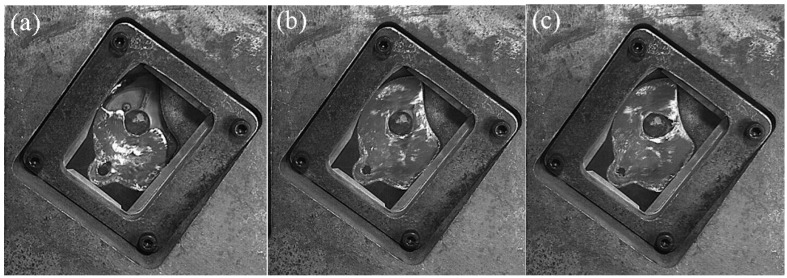
Flow pattern of A380 aluminum alloy melt with the fast shooting velocity of 0.66 m/s in the right window. (**a**–**c**): Filling status at different filling times.

Figure 15 shows the filling process at the fast injection speed of 0.66 m/s. The liquid passing through the cylinder cannot immediately merge because the cylinder affects the flow direction of the fluid, which causes the tail of the cylinder to be filled insufficiently. Figure 15a shows that the inner separation shear layer of the upstream cylinder had no ability to reattach to the outer surface of the downstream cylinder, so that the incoming flow from the ingate could pass through the gap between the two cylinders and directly flow upward to the R1 area. This flow pattern was similar to the induced separation flow pattern (IS) that was depicted by Sumner et al. [25]. This mode is also similar to the “Pattern IIB” mode that was defined by Gu and Sun [26]. Therefore, R1 was marked as the other zone, which was almost free from the near-tail interference.

R2 is the near-tail zone, which was located behind the upstream cylinder and was severely squeezed by the gap flow of the two cylinders. R3 is the final filling zone. In Figure 14b, there was a large enclosed space behind the downstream cylinder, which was in R3 and it is the last filling position.

**Figure 16:**
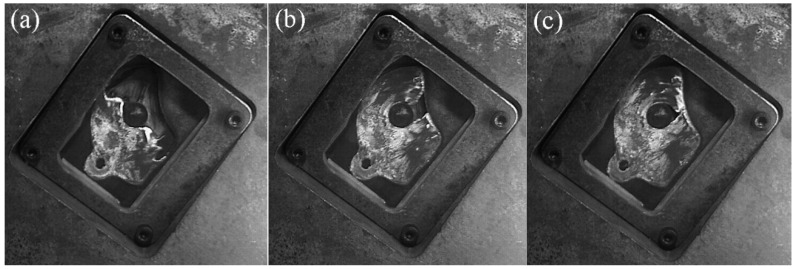
Flow pattern of A380 aluminum alloy melt with the fast shot velocity of 0.80 m/s in the right window. (**a**–**c**): Filling status at different filling times.

In Figure 16, the fast injection speed was 0.80 m/s and the flow pattern at this speed was almost the same as the flow pattern at 0.66 m/s. R1 was marked as other zone, R2 was the near-tail zone, and R3 was the final filling zone.

**Figure 17:**
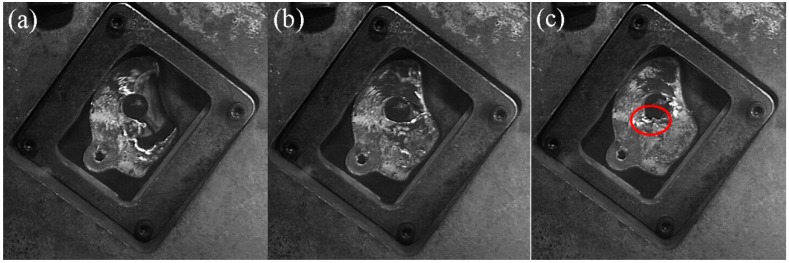
Flow pattern of A380 aluminum alloy melt with the fast shot velocity of 1.06 m/s in the right window. (**a**–**c**): Filling status at different filling times.

The filling pattern was different when the fast injection speed was 1.06 m/s (Figure 17). The velocity of the inflow through the two cylindrical gaps increased, the open space on the back of the downstream cylinder was squeezed to the R2 area along the outer surface of the downstream cylinder under the action of the wall surface (marked with red circle), and the R2 region became the final filling zone, due to the increase in the injection speed.

**Figure 18:**
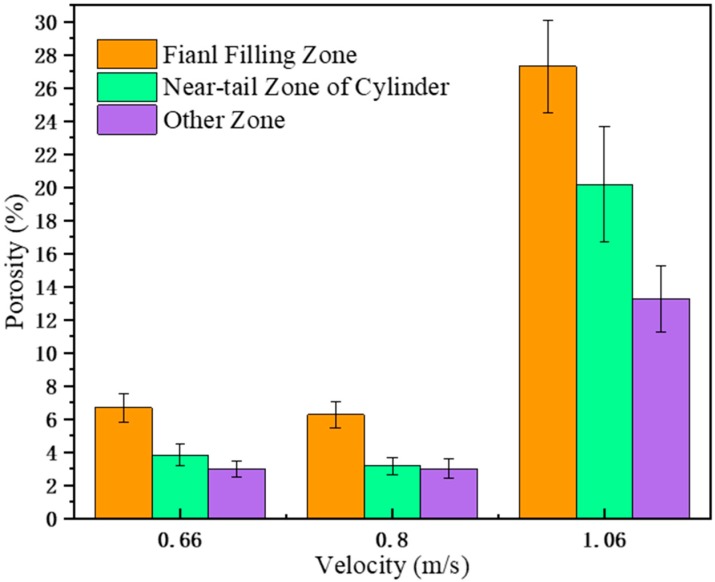
The porosity at different locations in the right window with different fast shooting velocities (0.66 m/s, 0.8 m/s, and 1.06 m/s).

Figure 18 shows the porosity at different positions of A380 aluminum alloy die casting in the right window at different fast injection speeds. The final filling zone had the highest porosity, followed by the near-tail zone of cylinder. The cylinder tail region had large negative pressure, and the maximum turbulence intensity was in the near-tail region, which caused a large amount of gas to be sucked in and trapped, hence the porosity was large.

**Figure 19:**
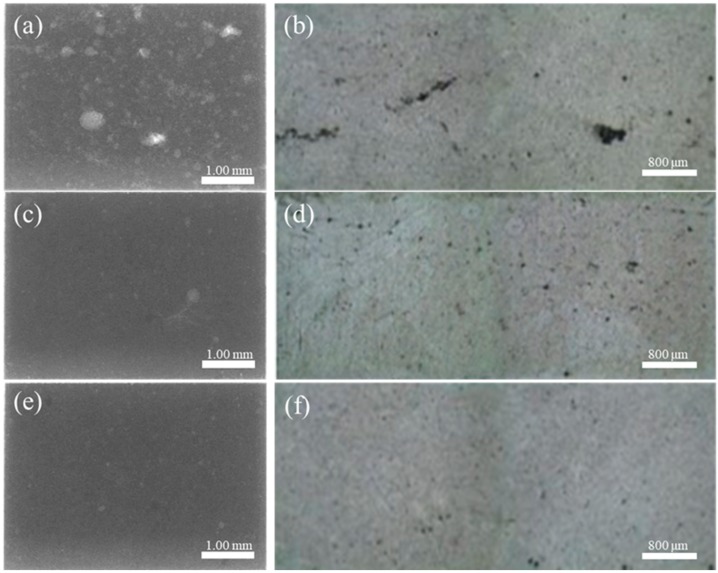
Comparison of porosity microstructure. (**a**) X-ray image of final filling zone; (**b**) OM image of final filling zone; (**c**) X-ray image of near-tail zone of cylinder;(**d**) OM image of near-tail zone of cylinder; (**e**) X-ray image of other zone; (**f**) OM image of other zone.

Figure 19 shows microstructures, and defects (porosity) of specimens from final filling zone, near-tail zone of cylinder, and other zone under the velocity of 0.8 m/s. The pores in the final filling zone are larger and more numerous, which has the same conclusion as the calculation.

## 4. Conclusions

In this study, we used a shock absorption tower and pure aluminum and A380 aluminum alloy to observe in real-time the die casting filling process with different fast injection speeds. We analyzed and predicted the locations and sizes of air entrapment defects combined with porosity measurements (calculation and microstructure analysis).

(1) Under similar injection velocity, the porosity of pure aluminum was significantly greater than the porosity of the aluminum alloy at each position. Pure aluminum had a large degree of fracture in the filling process, whereas the A380 aluminum alloy had a small degree of fracture and basically maintained a continuous state.
(2) The porosity of different regions was closely related to the flow state of the aluminum liquid. The highest porosity in the backflow zone, the second highest in the final filling zone, and the near-tail zone of the cylinder were determined from the filling process analysis and porosity calculation results. The final filling position changed as injection velocity changed.
(3) The pores in the flowback zone and final filling zone are larger and more numerous from the microstructure and pore defects shown in X-ray and OM images, which has the same conclusion as the porosity calculation.

## Figures and Tables

**Table 1:** Chemical composition of A380 aluminum alloy (wt.%).

Element	Si	Cu	Mg	Fe	Zn	Mn	Ni	Sn	Al
wt.%	9.146	3.140	0.104	0.154	0.141	0.103	0.101	0.100	REM

**Table 2:** Concrete parameters of the visualization experiment.

Experiment	Material	Pouring Temperature	Fast Shoot Speed
A1	Pure Aluminum	750 ± 10 °C	0.88 m/s
A2	Pure Aluminum	750 ± 10 °C	1.59 m/s
A3	Pure Aluminum	750 ± 10 °C	2.34 m/s
B1	A380	700 ± 10 °C	0.66 m/s
B2	A380	700 ± 10 °C	0.80 m/s
B3	A380	700 ± 10 °C	1.06 m/s

**Table 3:** Properties of borosilicate glass [20].

Properties	Value
Density	2.23 g/cm^3^
Hardness	6.5 Mohs’
Young’s Modulus	6680 N/mm^2^
Bending Strength	120–160 MPa
Poisson’s Ratio	0.20
Thermal Expansion Coefficient (20–350 °C)	32–35 × 10^−6^ cm/cm·°C
Thermal conductivity (20 °C)	0.82 W/m·°C
Specific Heat	820 J/kg·°C
**Chemical Composition (wt.%)**	**Value**
SiO_2_	81.0%
B_2_O_3_	12.5%
Al_2_O_3_	2.32%
Na_2_O+K_2_O	6.0%

**Table 4:** Physical parameters of pure aluminum and A380 aluminum alloy at the ingate under different temperature.

Material	Temperature (°C)	Density (kg/m^3^)	Surface Tension (10^−3^N/m)	Viscosity (10^−3^ kg/ms)	Ingate Velocity (m/s)	*Re*	*We*	*Oh* (×10^−4^)
Pure Aluminum	750	2363.47	836.90012	1.0837	17.6	95,960	2187	4.87
Pure Aluminum	710	2366.81	848.90546	1.1728	17.6	88,790	2159	5.23
A380	700	2482.5	742.06617	1.3761	16.0	72,102	2141	6.42
A380	660	2496.2	749.34843	1.5083	16.0	66,196	2132	6.98
